# Editorial: Gastrointestinal (GI) disorders and antioxidant therapeutics

**DOI:** 10.3389/fendo.2025.1588417

**Published:** 2025-03-26

**Authors:** Dipak Kumar Sahoo, Gagan B. N. Chainy, Albert E. Jergens

**Affiliations:** ^1^ Department of Veterinary Clinical Sciences, College of Veterinary Medicine, Iowa State University, Ames, IA, United States; ^2^ Department of Biotechnology, Utkal University, Bhubaneswar, Odisha, India

**Keywords:** gastrointestinal disorders, antioxidants, ROS, hormones, GI health, inflammatory bowel disease, chronic inflammatory enteropathy, colorectal cancer

Gastrointestinal (GI) disorders encompass diverse clinical conditions that impact the GI tract, such as inflammatory bowel disease (IBD), dyspepsia, and malignant tumors. Canine CIE exhibits several similarities with IBDs observed in human patients (1). Both acute and chronic GI disorders in humans and animal models are marked by a disruption in redox balance, which may result from increased production of reactive oxygen species (ROS) or weakened antioxidant defense systems (AODS). Oxidative stress (OS) denotes a disparity between oxidants and antioxidants, with a tendency towards the production of oxidants (2). This imbalance subsequently interferes with redox signaling and regulation, potentially leading to detrimental effects at the molecular level. Advancements in treatments for GI disorders, such as IBD, that are influenced by OS require greater comprehension of the cellular and molecular processes governed by reactive oxygen species (ROS). In the GI tract, oxidative stressors encompass infections and pro-inflammatory responses, which enhance the generation of ROS by stimulating the production of pro-inflammatory cytokines. Nuclear factor kappa B (NF-κB) and nuclear factor erythroid 2–related factor 2 (Nrf2) are two key signaling pathways in intestinal immune cells that govern various pathological processes, encompassing anti-inflammatory and antioxidant functions (3).

Hong et al. highlighted that Nrf2, mammalian target of rapamycin (mTOR), adenosine monophosphate-activated protein kinase (AMPK), and forkhead box protein O1 (FOXO1) could serve as significant targets that concurrently influence OS, inflammation, and autophagy, as studied in adipogenesis. The oxidative balance score (OBS) is frequently utilized to evaluate OS, offering a thorough assessment of dietary and lifestyle-related factors. Li et al. investigated the association between OBS and IBD. OBS demonstrated a negative correlation with IBD, particularly among female Crohn’s disease (CD) patients. This research highlights the importance of an antioxidant diet and lifestyle, potentially offering improved health for female patients with CD. The study conducted by Chang et al. indicates that increased exposure to antioxidants, evaluated through OBS (diet and lifestyle rich in antioxidants), could potentially reduce the incidence of colorectal cancer (CRC). A significant negative correlation was identified between OBS and the likelihood of developing CRC and its various subsites (proximal colon cancer, distal colon cancer, and rectal cancer). The correlation was especially evident among male CRC patients. Biomarkers are essential for the detection of diseases and for evaluating the effectiveness of treatments (4, 5). Serum albumin, uric acid, and the neutrophil count are biomarkers mediating the potential association between OBS and CRC.

**Figure 1:**
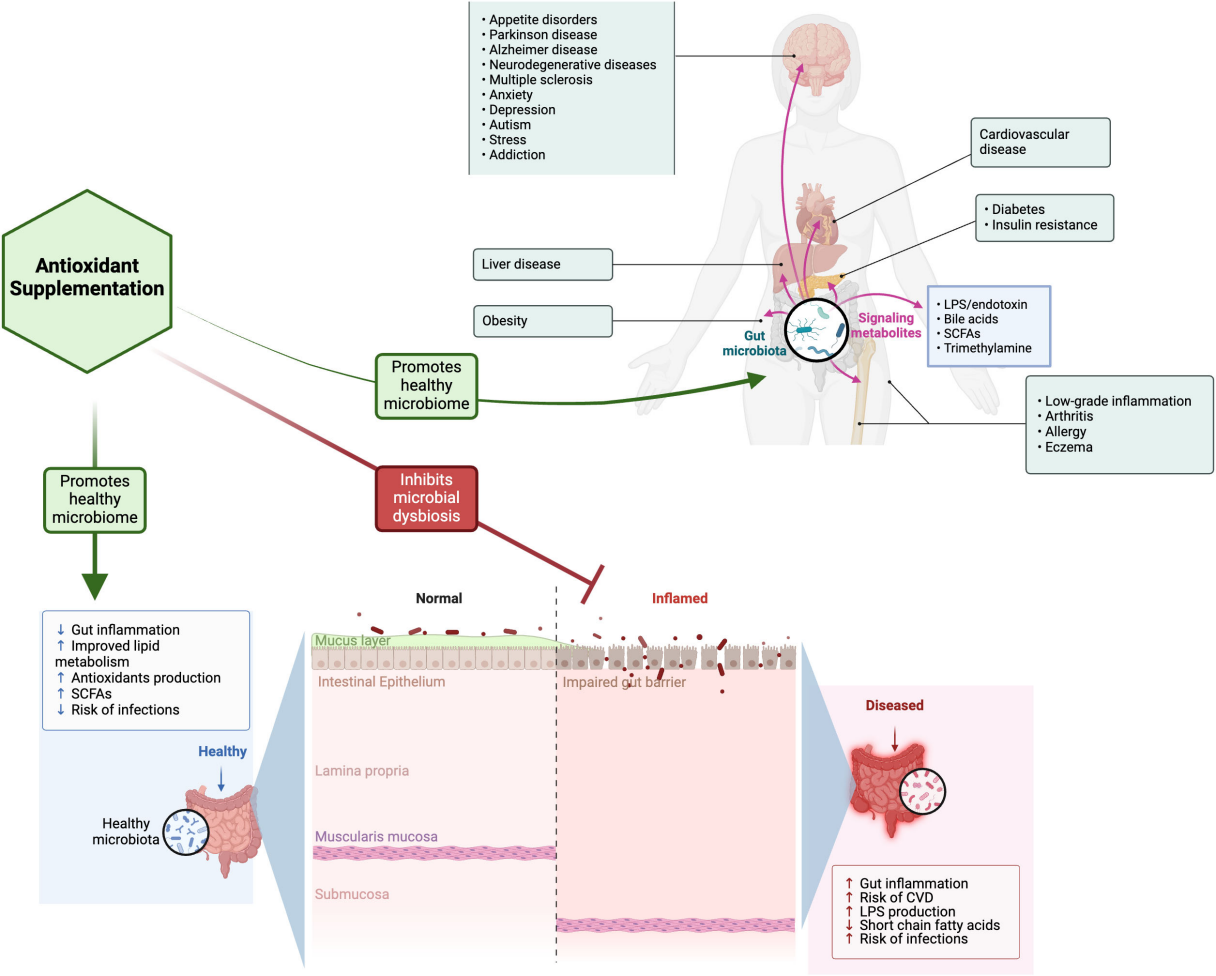
A schematic depiction of the influence of gut microbiota in nutrition and health with the potential protective effect of antioxidant supplementation in preserving gut health. This figure was created in BioRender (https://BioRender.com/u24c341). LPS, Lipopolysaccharide; CVD, cardiovascular disease; SCFAs, Short-chain fatty acids.

Antioxidants have been suggested as a possible alternative treatment to anti-inflammatory/immunomodulatory medications used to treat GI disorders (**Figure 1**). There are various types of antioxidants, each exhibiting distinct mechanisms of action and offering application to specific clinical applications (6). Natural antioxidant compounds can scavenge ROS and enhance the body’s antioxidant defense mechanisms (6–13), potentially inhibiting pro-oxidative enzymes aiding the treatment of IBD. When the antioxidant capacity of the damaged mucosa is diminished, the application of nontraditional therapies, including pharmaceuticals, natural or synthetic agents, hormones, and probiotics that neutralize reactive oxygen and nitrogen species, mitigate cellular damage (protein carbonylation, lipid peroxidation, and DNA modification) and enhance the function of antioxidant enzymes, are helpful when used in conjunction with anti-inflammatory medications or independently (14–17). Muro et al. provided a comprehensive overview of the involvement of OS in the pathophysiology of IBD, highlighting its diagnostic targets and exploring the potential use of antioxidant therapies to manage IBD. Markers of OS, including 8-hydroxy-2’-deoxyguanosine, malondialdehyde, and serum-free thiols, were increased in the blood and stool of patients and correlated with disease severity. Consequently, markers of OS can serve a dual purpose, aiding in both the diagnosis and the assessment of IBD treatment efficacy. The treatment of IBD can also focus on the use of antioxidants, such as glutathione, vitamin C, vitamin E, and N-acetylcysteine. In their review, Sahoo et al. examined a range of polyphenolic substances, including curcumin, resveratrol, quercetin, caffeic acid phenethyl ester, green tea flavonoids, luteolin, genistein, xanthohumol, alpinetin, silymarin, proanthocyanidins, anthocyanins and phenolic compounds such as thymol, alkaloids like berberine, storage polysaccharides including tamarind xyloglucan, and various phytochemicals represented by isothiocyanate sulforaphane and food/spices such as ginger, flaxseed oil, along with antioxidant hormones like melatonin, all of which target cellular signaling pathways to mitigate intestinal inflammation associated with IBD. He et al. evaluated the relationship between dietary carotenoid intake and soluble Klotho (S-Klotho) plasma levels in elderly adults. The overall consumption of carotenoids showed a positive correlation with plasma concentrations of S-Klotho among the elderly population, especially with ingestion of α-carotene, β-carotene, and lutein with zeaxanthin.

The gut microbiota (GM), which includes bacteria, archaea, fungi, protozoa, and viruses, significantly influences host health and disease (18, 19). The GM plays a crucial role in the intestinal epithelial barrier, aids in host metabolism, offers protection against pathogens, and impacts the development of the mucosal immune system (19). Numerous research findings illustrate the positive impacts of prebiotics, probiotics, postbiotics, and synbiotics on human and animal health, such as contributing to the maintenance of a healthy GI microbiota, enhancing the intestinal mucosal barrier, promoting immune tolerance, and helping regulate the pro-inflammatory response (18, 20–22). The underlying mechanisms of functional GI disorders encompass alterations in the gut microbiota/gut hormone axis, which play a crucial role in GI motility. Hormone receptors have been discovered in inflamed regions, demonstrating the ability to trigger both pro- and anti-inflammatory responses. Dai et al. conducted a comprehensive review of the pathogenesis of cardiovascular disease (CVD), addressing factors such as inflammatory response, OS, mitochondrial dysfunction, pyroptosis, ferroptosis, and dysbiosis of GM. Research indicates that the GM functions as a “microbial organ” influencing cardiovascular health, and the “gut-heart” axis presents a promising pathway for preventing and treating CVD. A significant number of potent compounds derived from traditional Chinese medicine (TCM) have been identified, including flavonoids, phenolic acids, stilbenes, saponins, anthraquinones, terpenoids, alkaloids, polysaccharides, which all demonstrate therapeutic effects on various CVDs. TCM can potentially address CVD through various mechanisms, such as antioxidant properties, anti-inflammatory effects, enhancement of mitochondrial function, prevention of cell death (including apoptosis, autophagy, pyroptosis, and ferroptosis), and modulation of GM. Clinical trials have demonstrated the effectiveness and safety of TCM in reducing the symptoms associated with CVD. Studies indicate that numerous TCM monomers can potentially enhance blood sugar and lipid levels and early renal function via the “gut-liver-kidney axis” while mitigating pathological damage to kidneys. Xu et al. demonstrated that TCM exhibits a protective influence on the kidneys through its interaction with farnesoid X receptor (FXR). TCMs have the potential to modulate the intestinal microbiota-bile acid (BA) axis, thereby slowing the progression of DKD. The distinctive benefits of Chinese medicine in addressing kidney tissue damage, managing glucose and lipid metabolism, lowering urinary protein levels, and mitigating the inflammatory response have been determined. In patients with DKD, there is a reduction in both probiotics and prebiotics, and supplementation of these elements can enhance glucose metabolism, restore the function of the intestinal mucosa, and reverse structural damage to renal tissue in individuals with DKD. In TCM, the combination of *Salvia miltiorrhiza* and *Astragalus membranaceus* is an effective prescription for the treatment of DKD (23). *A. membranaceus* and *S. miltiorrhiza* enhanced the prevalence of *Akkermansia*, *A. muciniphila*, *Lactobacillus*, and *L. murinus*. While *Akkermansia* and *A. muciniphila* exhibited a positive correlation with the synthesis of arachidonic acid metabolites, *Lactobacillus* and *L. murinus* exhibit a negative correlation with glycerophospholipid metabolism, suggesting that TCM may contribute positively to anti-inflammatory effects and lipid reduction through the enhancement of intestinal flora distribution. Another TCM, astragaloside IV (AS-IV), the active component in astragaloside, facilitates the release of Nrf2 from Kelch-like ECH-associated protein 1 (Keap1)-Nrf2 complex and counteracts mitochondrial dysfunction (24). Huang-Lian-Jie-Du decoction, a TCM formulation, has the potential to improve blood glucose metabolism in DKD rats through the modulation of the advanced glycation endproducts (AGE)/receptor for AGE (RAGE)/protein kinase B (Akt)/Nrf2 pathway while also lowering triglyceride and low-density lipoprotein cholesterol levels to safeguard kidney function (25). Mycobacterial species possess a powerful immunomodulatory capability, primarily due to their complex cell wall composition. Abdallah et al. conducted a study to explore the possible prophylactic anti-diabetic effects of heat-killed *Mycobacterium aurum* (HK-*MA*) in mice with streptozotocin (STZ)–induced diabetes. Prophylactic administration of three doses of HK-*MA* to diabetic mice led to a notable decrease in their blood glucose levels compared to the control diabetic mice. Prophylactic treatment of diabetic mice with HK-*MA* notably normalized their altered protein expression levels of liver uncoupling protein 2 (UCP2), lactate dehydrogenase, and skeletal muscle UCP3. HK-*MA* could lower hyperglycemia and may support its potential application as a dietary supplement for improving diabetes management. The decrease in hyperglycemia might be partially linked to an anti-inflammatory effect brought about by HK-*MA*. However, the potential influence of HK-*MA* on inflammation is still a hypothesis that necessitates additional research. In contrast, Shi et al. conducted a comprehensive review of the mechanisms by which the gut microbiome has tumor-promoting and potential anti-tumor effects. They outlined various bacterial therapeutic strategies for addressing tumors, encompassing natural and engineered anti-tumor approaches. In specific conditions, particular bacteria, notably anaerobes or parthenogenetic anaerobes, accumulate and proliferate within the tumor environment. This occurrence triggers a series of responses in the body that eventually lead to anti-tumor effects. The GI microbiome plays a significant role in tumor development through various mechanisms, such as the release of metabolic by-products, the induction of inflammatory cascades, the modulation of immune responses, and the alteration of microbial abundance and colonization sites. The metabolite indole-3-propionic acid, associated with quercetin, functions as an aryl hydrocarbon receptor (AhR) agonist. It reduces inflammatory responses in colonic epithelial cells, consequently hindering carcinogenesis and demonstrating anticancer effects. *Bifidobacterium* is classified among probiotics and has been linked to possible advantages in preventing IBD and CRC. The GM produces numerous metabolites that demonstrate anti-tumor effects by inhibiting the proliferation of tumor cells, regulating apoptosis, and suppressing inflammatory responses. It is important to highlight that butyrate, recognized as the most thoroughly researched short-chain fatty acids (SCFAs), has demonstrated a dual role, possessing the capacity to promote tumorigenesis and elicit anti-tumor effects. Butyrate demonstrates its ability to combat colon cancer effects by influencing Fas and p21 in animal tumor models and inhibiting enzymes associated with pro-carcinogenic activities in the intestine, including histone deacetylases. These actions reduce tumor cell proliferation and contribute to the inhibition of further CRC progression. Furthermore, butyrate plays a crucial role in preserving the integrity of the intestinal barrier, which is essential for the overall functioning of the intestines. A bidirectional two-sample Mendelian randomization (BTS-MR) study by Liu et al. thoroughly illustrated the intricate relationship between GM and thyroid dysfunction. This research underlines the importance of selecting more targeted probiotics to uphold thyroid–gut axis homeostasis, thereby aiding in the prevention, management, and reversal of thyroid dysfunction development. This BTS-MR study demonstrated that the genera *Intestinimonas*, *Eubacterium brachy* group, *Ruminiclostridium*, and *Ruminococcaceae* UCG004 were associated with an increased risk of decreased thyroid function. In contrast, the genera *Bifidobacterium* and *Lachnospiraceae* UCG008, along with the phyla Actinobacteria and Verrucomicrobia, exhibited protective effects.

Exploring natural products (26, 27) and the production of essential therapeutics through molecular farming (28) have garnered significant interest in recent years due to their potential therapeutic advantages and reduced adverse effects compared to synthetic medications. Considering the current body of scientific evidence, it seems possible that forthcoming therapies may incorporate antioxidants alongside conventional treatments or potentially serve as an alternative medical approach for humans and animals suffering from IBD. However, additional research is necessary before these antioxidants can be utilized in clinical practice.
